# Role of OPG/RANKL/RANK/TLR4 signaling pathway in sepsis-associated acute kidney injury

**DOI:** 10.1186/s12882-024-03648-1

**Published:** 2024-06-23

**Authors:** Xinrong Niu, Caihong Wang, Hui Li, Weilin Chen

**Affiliations:** Department of Critical Care Medicine, Xinjiang Uyghur Autonomous Region People’s Hospital, No. 91 Tianchi Road, Tianshan District, Urumqi, 830001 P.R. China

**Keywords:** Sepsis, Acute kidney injury, OPG/RANKL/RANK/TLR4 signaling pathway, Inflammatory response

## Abstract

**Background:**

Sepsis-associated acute kidney injury (SA-AKI) has high mortality rates. The osteoprotegerin (OPG)/receptor activator of nuclear factor-κB ligand (RANKL)/receptor activator of nuclear factor-κB (RANK)/Toll-like receptor 4 (TLR4) pathway and its potential role in SA-AKI pathogenesis remain to be fully understood. Herein, we addressed this issue using mouse models.

**Methods:**

An SA-AKI mouse model was established using the cecal ligation and puncture method (CLP). Mice were grouped into sham, CLP model, CLP + recombinant RANKL, and CLP + anti-RANKL groups. Serum creatinine (Scr) and blood urea nitrogen (BUN) levels were measured to assess kidney function. ELISA was used to detect serum IL-1β, TNF-α, and IL-6 levels. Real-time quantitative PCR and Western blot were used to detect the mRNA and protein expression levels of OPG, RANKL, RANK, and TLR4 in kidney tissues. HE staining was performed to evaluate the pathological changes.

**Results:**

The CLP model group showed higher levels of Scr and BUN, indicating impaired kidney function in SA-AKI, compared to the sham group. Treatment with recombinant RANKL in the CLP + recombinant RANKL group reduced Scr and BUN levels, while anti-RANKL treatment in the CLP + anti-RANKL group elevated their levels. Moreover, the CLP model group had significantly increased IL-1β, TNF-α, and IL-6 than the sham group, indicating elevated inflammation in SA-AKI. The CLP + recombinant RANKL group demonstrated decreased cytokine levels, whereas the CLP + anti-RANKL group showed an increase. Additionally, the histopathological evaluation revealed distinct kidney tissue damage in the CLP model group. Recombinant RANKL treatment reduced this damage, while anti-RANKL treatment exacerbated it. Mechanically, the mRNA and protein expression of RANKL were significantly decreased, while those of OPG, RANK, and TLR4 were significantly increased in the CLP model group and the CLP + anti-RANKL group. Interestingly, treatment with recombinant RANKL reversed these changes, as evidenced by significantly increased RANKL but decreased OPG, RANK, and TLR4.

**Conclusion:**

The OPG/RANKL/RANK/TLR4 pathway is involved in SA-AKI pathogenesis. Recombinant RANKL treatment attenuates the inflammatory response and kidney tissue damage in SA-AKI, possibly via regulating this pathway. This pathway shows promise as a therapeutic target for SA-AKI.

## Background

Sepsis is a life-threatening clinical syndrome that is prone to complications such as septic shock and acute kidney injury (AKI) [1]. It is a leading cause of mortality in intensive care unit patients [2], resulting in a considerable rise in mortality rates [3]. Sepsis-related acute kidney injury (SA-AKI) accounts for over 50% of clinical AKI cases [4]. In cases where sepsis is complicated by AKI, the mortality rate of the patient may reach 75% [5]. The primary treatment of SA-AKI is kidney replacement therapy. Kidney replacement therapy is limited to symptomatic treatment and is not correlated with the early diagnosis of AKI. Identifying new targets for the prevention and treatment of SA-AKI will effectively reduce the mortality rate of critically ill patients. Therefore, understanding the pathogenesis of SA-AKI and exploring novel treatment strategies are of particular significance [6].

Osteoprotegerin (OPG) is a secreted protein involved in the regulation of bone resorption, and it is distributed in organs including the heart, kidneys, liver, bones, and blood vessels in the human body. The receptor activator of nuclear factor-κB ligand (RANKL) is a type II transmembrane protein, while the receptor activator of nuclear factor-κB (RANK) is a type I transmembrane protein that belongs to the tumor necrosis factor receptor superfamily. RANKL is presently the sole known ligand that binds to the RANK membrane receptor, leading to receptor trimerization and subsequently initiating the activation of TNF receptor-associated factors (TRAF) and downstream signaling pathways [7]. OPG, also identified as the decoy receptor for RANKL, competes with RANKL for binding and the interaction of these three components constructs a regulatory system [8]. The involvement of the OPG/RANKL/RANK pathway in pathological angiogenesis, cell survival, bone metabolism, prevention and treatment of acute inflammatory diseases, development of breast epithelial cells, immune function, and cancer has been reported [9, 10]. However, there is limited information on whether this signaling pathway contributes to the development of SA-AKI through participation in the imbalance of inflammatory reactions and endothelial dysfunction.

Toll-like receptor 4 (TLR4) is a natural immune receptor, and the excessive activation of TLR4 triggers the production of various inflammatory factors, playing a crucial role in the induction of inflammatory responses [11, 12]. It is reported that the binding of RANKL to RANK can inhibit the activation of TLR4 in macrophages, promote the binding between TRAF6 (a key activator of TLR4) and RANK, and affect upstream events of nuclear factor-kappa B activation, thereby reducing the expression of pro-inflammatory factors [10].

Herein, our study investigated whether it is possible to mitigate the inflammatory response of SA-AKI and improve SA-AKI by regulating the OPG/RANKL/RANK/TLR4 signaling pathway. We established an SA-AKI model through the cecal ligation and puncture (CLP) method, administered recombinant RANKL and anti-RANKL pretreatment, and detected the levels of inflammatory factors in four groups. The mRNA and protein expression levels of OPG, RANKL, RANK, and TLR4 were measured, while kidney tissue damage was observed. Our findings may provide experimental evidence for the development of therapeutic targets for SA-AKI.

## Methods

### Study animals

Twenty-four healthy male C57BL/6 mice, 8–10 weeks old with a body weight of 20–25 g, were purchased from the Animal Experimental Center of Xinjiang Medical University (animal license number: SCXK (Xin) 2018-0001). The mice were acclimated in the animal room with a constant temperature (22–24 °C) and a light/dark cycle (12 h) for 3 days before the experiment. They were fasted for 8 h before the experimental procedure but had access to water. All methods were performed following the relevant guidelines and regulations. The experimental protocol was reviewed and approved by the Experimental Animal Ethics Committee of the Xinjiang Medical University (Approval No.: IACUC-20230217-18). The study is reported in accordance with ARRIVE guidelines.

### Model establishment and animal grouping

Mice were randomly divided into the sham group, CLP model group, CLP + recombinant RANKL group, and CLP + anti-RANKL group, with 6 mice in each group. The SA-AKI model was established using the CLP method [13]. Briefly, after anesthetizing with 3% pentobarbital sodium (1 mL/kg) by intraperitoneal injection, a 1–1.5 cm incision was made along the midline of the lower abdomen. The cecum was ligated with a size 4 surgical suture 1 cm away from the end of the cecum. An 18-gauge needle was used to puncture the ligation site on both sides of the cecal wall twice, and a small amount of feces was squeezed out. After confirming the patency of the puncture site, the cecum was returned to the abdominal cavity, and the abdomen was closed in layers. The sham group only underwent laparotomy and closure of the abdomen without cecal ligation and puncture. The CLP + recombinant RANKL group and CLP + anti-RANKL group were intraperitoneally injected with recombinant RANKL (5 µg per mouse; Abclonal, Wuhan, China) and anti-RANKL (200 µg per mouse; Bio X cell, West Lebanon, NH, USA), respectively, 2 h before modeling, while the sham group and CLP model group were intraperitoneally injected with an equal amount of sterile water for injection. After the surgical procedure, the mice were immediately given 30 ml/kg of normal saline subcutaneously on the back of the neck for fluid resuscitation and kept warm while waiting for anesthesia recovery.

### Sample collection

At 24 h after modeling, blood was taken from the orbital vein, and serum was obtained after centrifugation (3,000 r/min, 15 min). The serum was stored at -80 °C until further analysis. After blood collection, the mice were euthanized by cervical dislocation. The right kidney tissue was collected and stored at -80 °C until further analysis. The left kidney tissue was also collected and fixed in 4% paraformaldehyde.

### Detection of serum creatinine (scr) and blood Urea Nitrogen (BUN)

Scr level was determined using the creatinine oxidase method, and BUN level was determined using the urease method, strictly following the instructions of the creatinine assay kit and urea nitrogen assay kit (Nanjing Jiancheng Bioengineering Institute, China).

### ELISA

The levels of IL-1β, TNF-α, and IL-6 in the serum were measured with corresponding ELISA kits (ELK Biotechnology CO., LTD, Wuhan, China), following the kit instructions.

### Real-time quantitative PCR (RT-qPCR)

Total RNA was extracted from the right kidney tissues. The cDNA was obtained after reverse transcription. The RT-qPCR was performed. The mRNA expression levels of *OPG*, *RANKL*, *RANK*, and *TLR4* were calculated using the 2^−ΔΔCT^ method.

### Western blotting

The right kidney tissues were subjected to tissue lysis and then the total protein was extracted after centrifugation at 12,000 rpm for 5 min at 4 °C. The protein concentration was detected with the BCA method. After electrophoresis separation, the proteins were transferred to the membrane, which was blocked with 5% skim milk for 2 h. Subsequently, the membrane was incubated overnight at 4 °C with the primary antibodies against OPG (1:1000; Affinity Bioscience, OH, USA), RANKL (1:3000; Proteintech Group, Inc, Wuhan, China), RANK (1:500; Abcam, Cambridge, UK), TLR4 (1:500; Proteintech Group, Inc), and β-actin (1:10000) (Tiandeyue (Beijing) Biotechnology Co., Ltd, China). The next day, the membrane was washed with TBST buffer and then incubated with the horseradish peroxidase-conjugated sheep anti-rabbit or anti-mouse secondary antibody (1:10000; Wuhan Aspen Biotechnology Co., Ltd, China). Lastly, the relative expression levels of the target proteins were analyzed using Image J and normalized to β-actin.

### Hematoxylin and Eosin (HE) staining

The left kidney tissue was fixed in 4% paraformaldehyde for 24 h, rinsed in running water for 2 h, dehydrated in graded ethanol, cleared, immersed in paraffin for 1 h, embedded, sectioned, stained with HE, mounted with neutral gum, and observed under a microscope for morphological changes. The histopathological scoring criteria were as follows: no apparent lesions (0 points), interstitial congestion or glomerular atrophy (1 point), tubular epithelial necrosis (1 point), tubular lumen dilation (1 point), and interstitial inflammatory cell infiltration (1 point), with a total score of 5 points [14].

### Statistical analysis

Data analysis was performed using SPSS 26.0 and GraphPad Prism 9.0 software. Normally distributed quantitative data are expressed as mean ± standard deviation. The t-test was used for comparisons between two groups, and analysis of variance was used for comparisons among multiple groups. A P value of < 0.05 was considered statistically significant.

## Results

### Levels of Scr and BUN

**Fig. 1:**
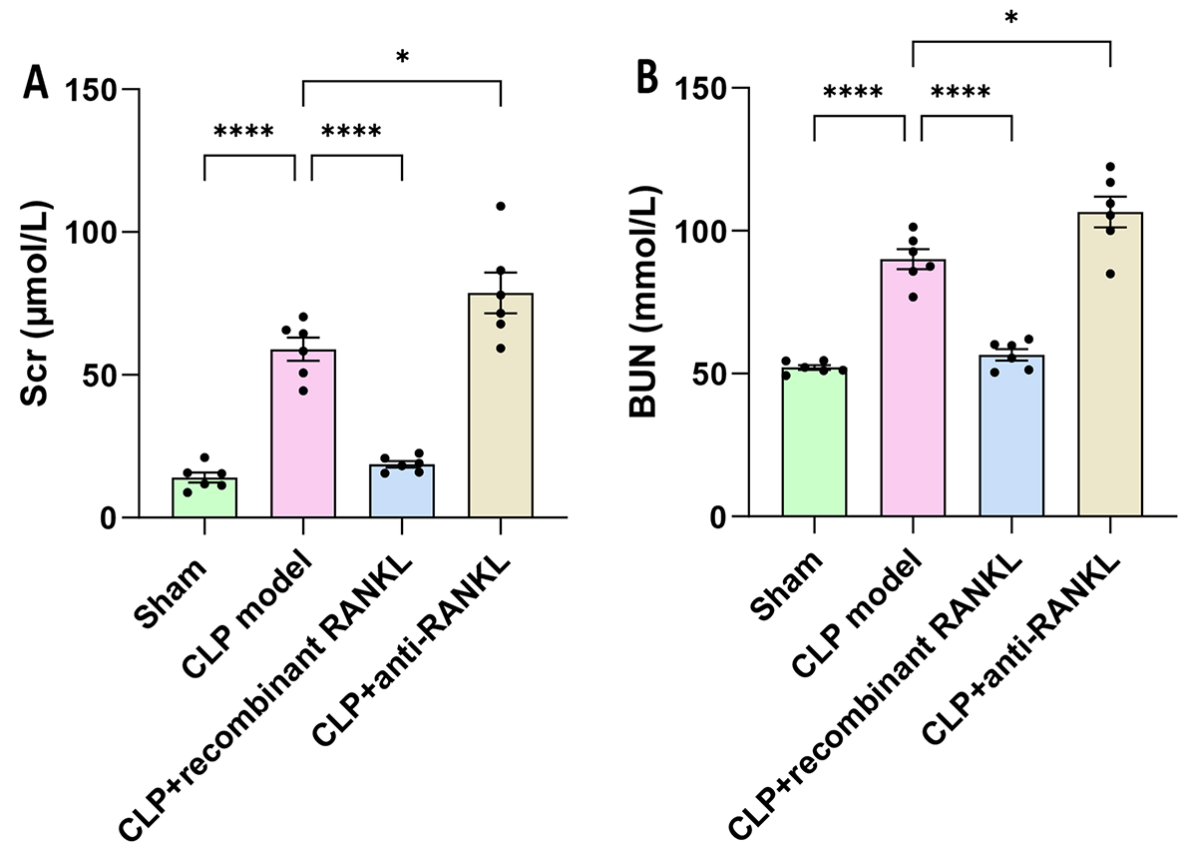
Levels of Scr and BUN in each group. (**A**) Scr level in each group. (**B**) BUN level in each group. ^*^P < 0.05, ^****^P < 0.0001

To investigate whether OPG/RANKL/RANK is related to the occurrence and development of SA-AKI, the levels of Scr and BUN were detected. The results revealed that the CLP model group showed a significant increase in the levels of Scr (Fig. 1A) and BUN (Fig. 1B) compared to the sham group (*P* < 0.05). In contrast to the CLP model group, the CLP + recombinant RANKL group exhibited a significant decrease in Scr and BUN levels, whereas the CLP + anti-RANKL group showed a significant increase (*P* < 0.05). This indicates the involvement of OPG/RANKL/RANK in the occurrence and development of SA-AKI.

### Changes in serum cytokine levels

**Fig. 2:**
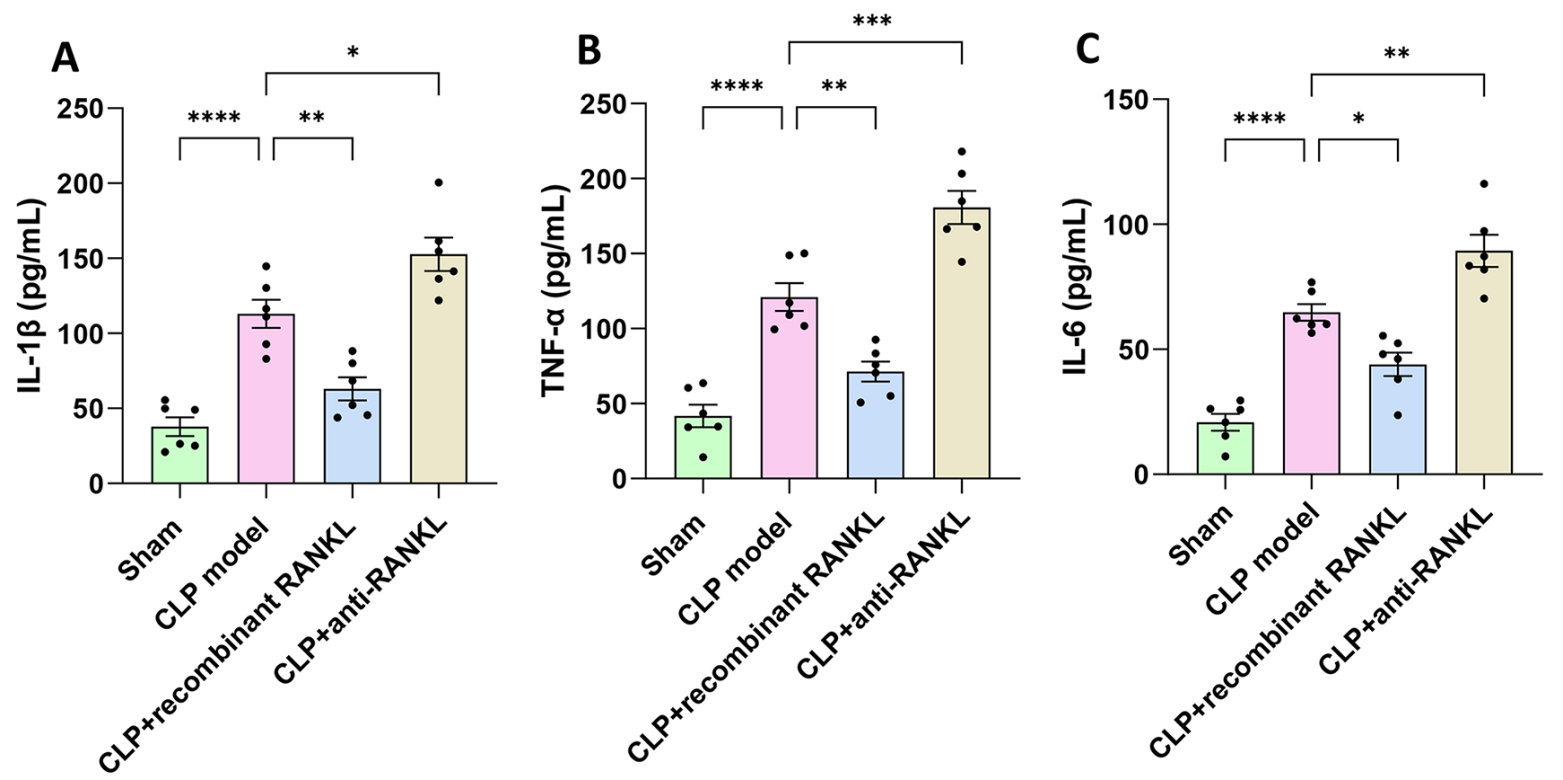
Serum levels of cytokines in each group. Cytokine levels were detected with ELISA. (**A**) IL-1β level in serum. (**B**) TNF-α level in serum. (**C**) IL-6 level in serum. ^*^P < 0.05, ^**^P < 0.01, ^****^P < 0.0001

We further detected the serum levels of IL-1β, TNF-α, and IL-6 by using ELISA to examine whether inflammatory response is involved in the occurrence and development of SA-AKI. Compared with the sham group, the serum levels of IL-1β (Fig. 2A), TNF-α (Fig. 2B), and IL-6 (Fig. 2C) in the CLP model group were significantly increased (*P* < 0.05). Compared with the CLP model group, the levels of the above factors were significantly decreased in the CLP + recombinant RANKL group but were significantly increased in the CLP + anti-RANKL group (*P* < 0.05). This suggests the involvement of inflammatory response in the pathogenesis of SA-AKI.

### The expression levels of *OPG*, *RANKL*, *RANK*, and *TLR4* mRNA in kidney tissues

**Fig. 3:**
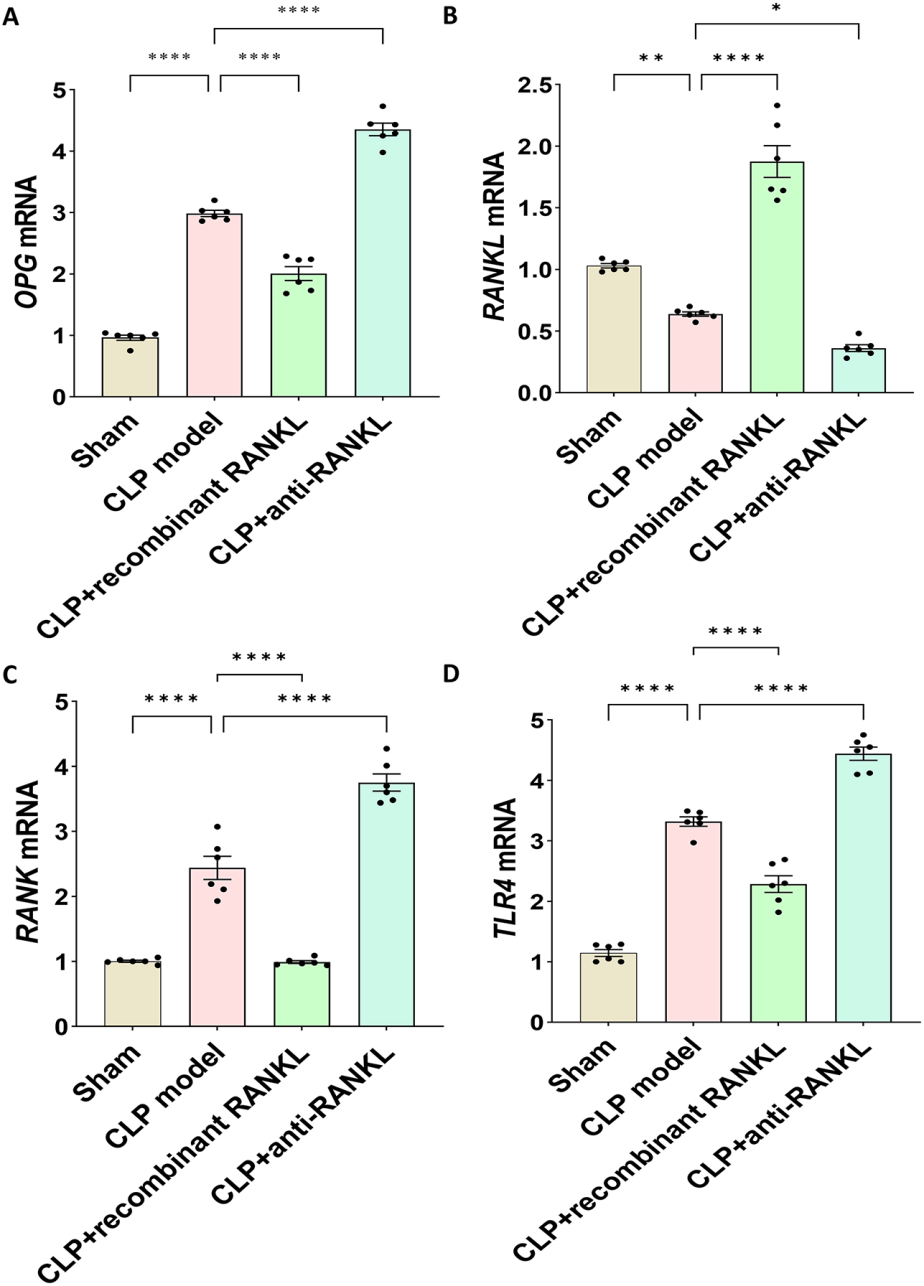
The expression levels of *OPG*, *RANKL*, *RANK*, and *TLR4* mRNA in kidney tissues. RT-qPCR detected the mRNA expression levels of each gene. (**A**) *OPG* mRNA. (**B**) *RANKL* mRNA. (**C**) *RANK* mRNA. (**D**) *TLR4* mRNA. ^*^P < 0.05, ^**^P < 0.01, ^****^P < 0.0001

To determine the expression of OPG/RANKL/RANK/TLR4 in SA-AKI, we conducted RT-qPCR. In kidney tissues, the mRNA expression levels of *OPG* (Fig. 3A), *RANKL* (Fig. 3B), *RANK* (Fig. 3C), and *TLR4* (Fig. 3D) differed among groups. Specifically, *RANKL* mRNA expression was significantly decreased in the CLP model group compared to the sham group, while *OPG*, *RANK*, and *TLR4* mRNA expression was significantly increased (*P* < 0.05). Moreover, the CLP + recombinant RANKL group exhibited contrasting results compared to the CLP model group for these indicators (*P* < 0.05). Additionally, the CLP + anti-RANKL group demonstrated increased levels of *OPG*, *RANK*, and *TLR4* mRNA while decreasing *RANKL* mRNA expression compared to the CLP model group (*P* < 0.05).

### The expression levels of OPG, RANKL, RANK, and TLR4 proteins in kidney tissues

**Fig. 4:**
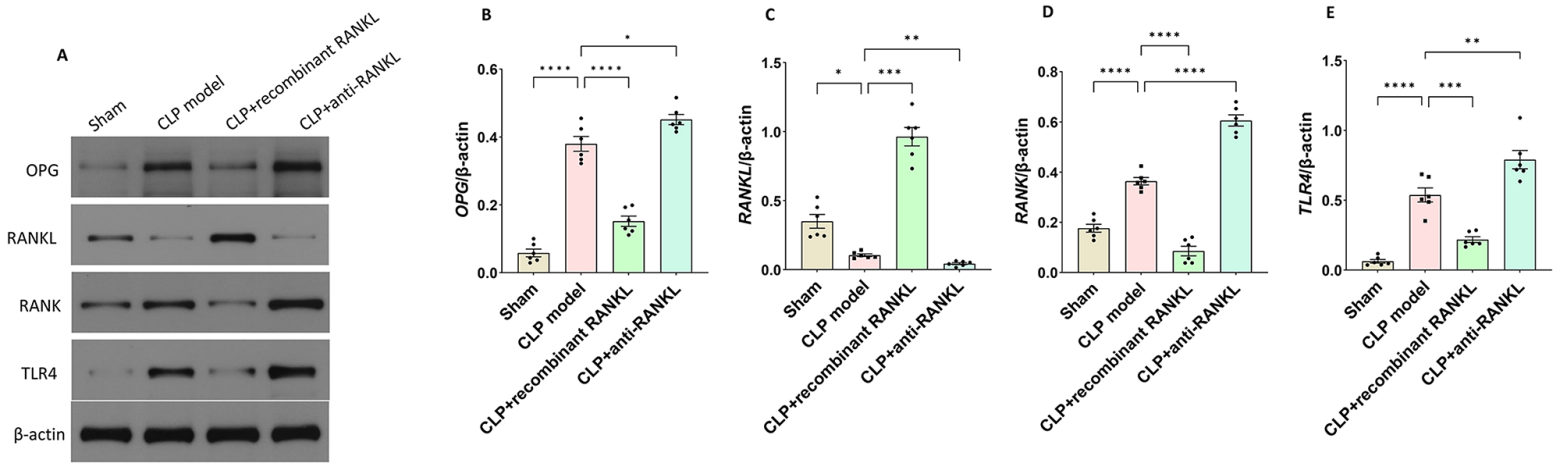
The expression levels of OPG, RANKL, RANK, and TLR4 protein in kidney tissues. Western blot detected the protein expression levels in each group. (**A**) Representative Western blot results. (**B**) Relative level of OPG. (**C**) Relative level of RANKL. (**D**) Relative level of RANK. (**E**) Relative level of TLR4. ^*^P < 0.05, ^**^P < 0.01, ^****^P < 0.0001

We further performed the Western blot to measure the expression levels of OPG, RANKL, RANK, and TLR4 proteins in kidney tissues (Fig. 4). Compared with the sham group, the protein expression of RANKL (Fig. 4A and C) in the kidney tissues of the CLP model group was significantly decreased, while the protein expression of OPG (Fig. 4A and B), RANK (Fig. 4A and D), and TLR4 (Fig. 4A and E) was significantly increased (*P* < 0.05). There were significantly higher levels of RANKL, but significantly lower levels of OPG, RANK, and TLR4 proteins in the CLP + recombinant RANKL group than in the CLP model group (*P* < 0.05). On the contrary, the CLP + anti-RANKL group had higher levels of OPG, RANK, and TLR4 proteins but lower RANKL than the CLP model group (*P* < 0.05). This data is consistent with those of RT-qPCR, indicating the involvement of OPG/RANKL/RANK/TLR4 in the occurrence and development of SA-AKI.

### Histopathological changes in the kidney tissues

**Fig. 5:**
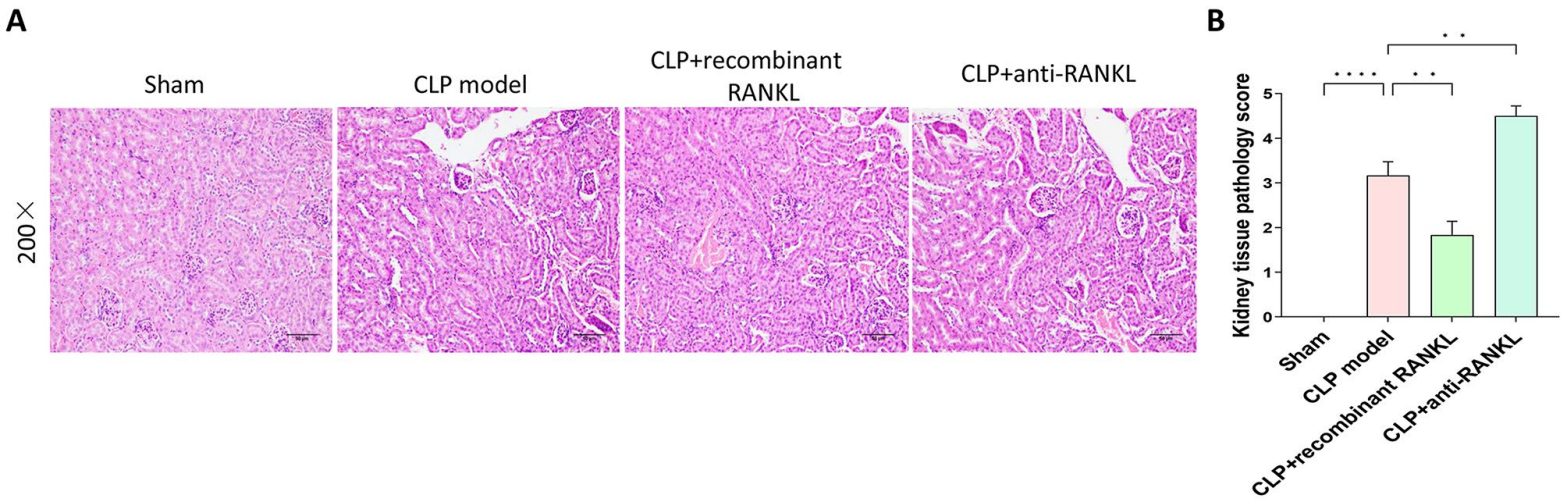
Pathological changes of the kidney tissues in each group. H&E staining evaluated the pathological changes in the kidney tissues. (**A**) Representative H&E staining images (200 × magnification). (**B**) The histopathological scores of each group. ^**^P < 0.01, ^****^P < 0.0001

The histopathological changes in the kidney tissues were detected with HE staining and the results are presented in Fig. 5. The histopathological scores of each group were calculated and compared. Following 24 h of modeling, no evident abnormalities were detected in the kidney tissue of the sham group (Fig. 5A). The kidney tissue structure in the CLP model group exhibited damage, including partial ischemic contraction of the kidney glomerulus, enlargement of the capsular space, necrosis, and shedding of kidney tubular epithelial cells into the tubular lumen, tubular dilation, kidney interstitial edema, infiltration of inflammatory cells, and a markedly higher histopathological score than the sham group. In the CLP + recombinant RANKL group, the extent of kidney tissue damage was decreased compared to the CLP model group, resulting in a significantly lower histopathological score (*P* < 0.05) (Fig. 5B). Conversely, the CLP + anti-RANKL group exhibited increased kidney tissue damage compared to the CLP model group, leading to a significantly higher histopathological score (*P* < 0.05). These results demonstrate that recombinant RANKL attenuates kidney tissue damage, whereas the use of anti-RANKL results in an exacerbation of the damage.

## Discussion

Sepsis has a rapid onset, quick progression, and poor prognosis, and can involve multiple organs throughout the body (such as blood vessels, kidneys, lungs, liver, heart, and brain) [15]. According to research, the mortality rate of sepsis in China is as high as 67 per 100,000 [16]. The mortality rate of sepsis is closely related to the development of organ dysfunction, with AKI being the most common, most severe, and highest mortality complication [17]. AKI progresses rapidly, is highly critical, and has a high degree of disability and fatality [18]. In this study, a sepsis mouse model was established using the CLP method. At 24 h after the surgery, the mice showed signs of lethargy, reduced activity, increased heart rate, and decreased urine output in the CLP group (data not shown). Laboratory tests showed a significant increase in Scr, BUN, IL-1β, TNF-α, and IL-6 and aggravated pathological changes in the kidneys of the CLP group, indicating the successful establishment of a mouse model with SA-AKI.

OPG, a member of the tumor necrosis factor superfamily, has been shown to directly participate in the inflammatory process associated with inflammatory bowel disease [19]. In AKI secondary to liver cirrhosis, serum OPG levels increase as the injury progresses, thereby exacerbating its severity [20]. Additionally, two recent clinical studies have identified OPG as a predictor of mortality in patients with systemic inflammatory response syndrome in intensive care units [21, 22]. Moreover, circulating OPG significantly increases in the serum of patients with SA-AKI, leading to an exacerbation of the inflammatory cascade reaction during SA-AKI [22]. Consistently, we found that the levels of inflammatory factors and the mRNA and protein expression of OPG in the serum of the CLP group mice were significantly higher than those in the Sham group. Moreover, there was inflammatory cell infiltration in the CLP group. After pretreatment with recombinant RANKL and anti-RANKL, the mRNA and protein expression of OPG showed a decrease and increase, respectively, and the degree of kidney tissue injury showed a reduction and exacerbation. These findings indicate that OPG is involved in the process of SA-AKI and may exacerbate SA-AKI by increasing the inflammatory cascade reaction.

It has been reported that RANKL plays a key role in many immune-inflammatory disorders [23]. Upon binding of RANKL to the receptor RANK, it can exert potent anti-inflammatory effects [24]. External application of recombinant RANKL protein can increase the expression of IL-10, thereby exerting anti-inflammatory effects [25]. The presence of RANKL in the nervous system can reduce neuronal death caused by microglia during stroke. The augmentation of RANKL/RANK signaling with recombinant RANKL protein can downregulate inflammatory factors, thereby alleviating the inflammatory response after stroke [26]. Similarly, in a mouse model pretreated with LPS, soluble RANKL was identified in the serum, and the level of RANKL was significantly reduced [27]. It was also found that RANKL inhibited the production of pro-inflammatory cytokines in macrophages [27]. In this study, we obtained similar results. Compared with the Sham group, the mRNA and protein expression of RANKL in the CLP group were significantly downregulated. The level of inflammatory factors was significantly increased, and the kidney tissue structure was damaged. In contrast, in the group pre-treated with recombinant RANKL, there was upregulated expression of RANKL at mRNA and protein levels. This may be attributed to the exogenous supplementation of RANKL. However, the underlying mechanism remains to be further determined. Moreover, the inflammatory response and kidney pathological damage were significantly reduced. Conversely, the anti-RANKL pre-treatment group showed an aggravation compared to the CLP group, suggesting that recombinant RANKL enhances the signaling of RANK, thereby alleviating the inflammatory response and kidney function damage of SA-AKI. Therefore, the mechanism by which RANKL exerts anti-inflammatory effects after binding to RANK may be related to the downregulation of OPG expression.

TLR4 is an important member of the TLR family, which triggers a cascade reaction upon binding to lipopolysaccharide, promoting the production of TNF, IL-1β, IL-6, and other pro-inflammatory cytokines, thereby initiating a pro-inflammatory immune response [28–30]. Inhibiting TLR4 signaling can significantly reduce the severity of SA-AKI in mice [31, 32]. The results of this study revealed a significant increase in TLR4 mRNA and protein expression in the CLP group, accompanied by significant inflammation, indicating that TLR4 is involved in the occurrence and development of SA-AKI. Therefore, inhibiting TLR4 signaling can mitigate the inflammatory response, thereby improving SA-AKI. Interestingly, the mRNA and protein expression levels of TLR4 in the recombinant RANKL pretreatment group were significantly downregulated, and the levels of inflammatory factors and the degree of kidney injury were significantly reduced compared to the CLP group. However, the results were abolished in the anti-RANKL pretreatment group and were more significant than the CLP group. These findings indicate that the combination of RANKL with RANK can inhibit the activation of TLR4, thereby alleviating the inflammatory response during SA-AKI and reducing the degree of kidney injury.

There are some limitations in this study. For example, due to limited funding, we did not comprehensively assess the involvement of inflammasome NLRP3, cell death mechanisms, or macrophages in SA-AKI, nor did we explore their relationship with the OPG/RANKL/RANK/TLR4 signaling pathway. Further studies are warranted.

**Fig. 6:**
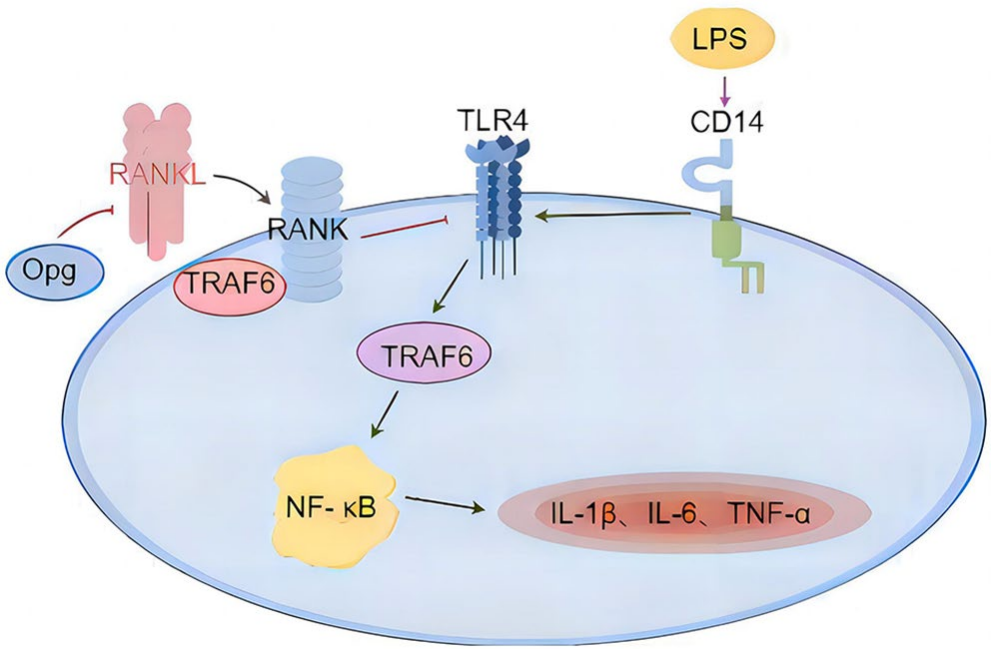
A schematic diagram illustrating the OPG/RANKL/RANK/TLR4 signaling pathway. RANKL competes with OPG for binding to RANK. Upon binding of RANKL to RANK, it triggers the interaction of RANK with TRAF6, leading to the suppression of the TLR4 signaling pathway, the activation of which is initiated upon the binding of LPS with CD14. This in turn inhibits the activation of the NF-κB signaling pathway and the subsequent release of inflammatory mediators

## Conclusion

In summary, our results demonstrate that RANKL plays an important role in preventing sepsis-associated AKI. Its mechanism may involve increasing the signal transduction after RANKL binds to RANK and reducing the expression of OPG and TLR4, thereby inhibiting the inflammatory response, reducing kidney tissue damage, and alleviating kidney dysfunction (Fig. 6). Therefore, the OPG/RANKL/RANK/TLR4 signaling pathway may become a potential therapeutic target for SA-AKI, providing a theoretical basis and intervention potential for clinical diagnosis and treatment. In the future, we will further investigate the mechanisms underlying the role of the OPG/RANKL/RANK/TLR4 signaling pathway in SA-AKI.

## Data Availability

The datasets used and/or analyzed during the current study are available from the corresponding author on reasonable request.

## Abbreviations

AKI: Acute kidney injury
SA-AKI: Sepsis-related acute kidney injury
OPG: Osteoprotegerin
RANKL: Receptor activator of nuclear factor-κB ligand
RANK: Receptor activator of nuclear factor-κB
TRAF: TNF receptor-associated factors
TLR4: Toll-like receptor 4
CLP: Cecal ligation and puncture
